# Supplementary material for: Explicit and implicit attitudes toward smoking: Dissociation of attitudes and different characteristics for an implicit attitude in smokers and nonsmokers
Source: PLoS One. 2022 Oct 10;17(10):e0275914. doi: 10.1371/journal.pone.0275914 (PMC9550055; doi:10.1371/journal.pone.0275914)
Supplement: Attachment — Submitted filename: Response_to_Reviewers.docx [file pone.0275914.s002.docx]

**Response to reviewers**

**[Reviewer #1]**

*This is an excellent article which summarize the main topics of the study, arguments, positions, analysis and findings. In my point of view, the paper provides new knowledge of this field of research and it can be published with no additional revisions.*

**Response:**

**We thank the reviewer for carefully reading our manuscript and providing positive comments.**

**[Reviewer #2]**

*I appreciate the opportunity given to review this interesting and informative paper on explicit and implicit attitudes toward smoking: Dissociation of attitudes and different characteristics for an implicit attitude in smokers and nonsmokers.*

*The focus of the study is clearly defined in terms of what aspects of attitudes toward smoking are under observation (implicit and explicit attitudes). The originality of the research is unquestionable as it differs from other literature on the differences in attitudes toward smoking between smokers and nonsmokers since it emphasizes the role of both implicit and explicit attitudes. The study also shows adequate knowledge and understanding of relevant literature on the concept of attitudes toward smoking.*

*Variables used in the study are well defined. Data analysis was properly done and results were well presented. The results from the sample used in the study presented adds to the growing literature on the concept.*

*The discussion section is well written. The authors make adequate references to other studies that relate to the outcome of their study. Also, the sentences are clearly expressed and readable.*

*However, there were a few grammatical errors identified. For instance, the authors wrote “hus” instead of “Thus” in line 70. It is recommended that the authors would revise the manuscript to check for grammatical errors.*

*Again I am grateful for the opportunity to review this manuscript and hope to see it in print.*

**Response:**

**We thank the reviewer for carefully reading our manuscript and providing positive comments. Following the suggestions of Reviewer #2 and the Academic Editor, we have revised the manuscript and checked for grammatical errors. The changes are shown in red font in the main manuscript.**

**[Journal Requirements and Editor comments]**

**We thank the editor for carefully reading our manuscript and identifying grammatical errors. We revised the manuscript and asked Editage to proofread it. The changes are shown in red font in the main manuscript.**

*1. Please ensure that your manuscript meets PLOS ONE's style requirements, including those for file naming.*

**[Response]**

**We revised our manuscript to meet PLOS ONE’s style requirements.**

*2. In your Data Availability statement, you have not specified where the minimal data set underlying the results described in your manuscript can be found. PLOS defines a study's minimal data set as the underlying data used to reach the conclusions drawn in the manuscript and any additional data required to replicate the reported study findings in their entirety. All PLOS journals require that the minimal data set be made fully available. For more information about our data policy, please see http://journals.plos.org/plosone/s/data-availability.*

**[Response]**

**We have provided the minimal data set underlying the results described in our manuscript as supporting information files. Please confirm it.**

*3. Please review your reference list to ensure that it is complete and correct.*

**[Response]**

**We have checked our reference list. We removed reference No.23 and replaced it relevant reference. The changes are shown in red font in the main manuscript.**

*Additional Editor Comments:*

**[Response]**

**We revised the manuscript with respect to all of the following points and asked Editage to proofread it. The changes are shown in red font in the main manuscript. The line numbers of the revised text are indicated below in bold text.**

*Line 31; rephrase...among undergraduate and graduate health sciences students. (or use "a" instead of "the"...health sciences course.* **(Lines 31–32)**

*Line 63; complaining vs complain* **(Line 67)**

*Line 70; thus vs hus* **(Line 74)**

*Line 79-81; the sentence needs to be reviewed. It is not clear what the pronoun "it" refers to in this context. Does it refer to "recent studies"? in which case "they" would be more appropriate than "it". Or, does it refer to "implicit process"? in which case it would be more appropriate to say "measure" as opposed to "measured" in this context.* **(Line 84)**

*Line 84; an "a" is missing before smoking cessation intervention* **(Line 88)**

*Line 91-93; Need to clarify what is meant by trials in this context. It is not intuitive that the authors are referring to repeated testing using the tool as this is not explained prior to using the term. Since the strengths of the tool is being discussed, the word trial can imply a clinical trial.* **(Line 95–96)**

*Line 109-111;The sentence is not clear and needs to be rephrased. ("the both attitudes" is not grammatically correct)* **(Line 113)**

*Line 114; The sentence is not clear and needs to be rephrased. (would not be significant vs would be not significant)* **(Line 118)**

*Line 347; is plausible vs was plausible* **(Line 353)**

*Line 357-358; Rephrase for clarity* **(Line 363–365)**

*Line 361; is plausible vs was plausible* **(Line 367)**

*Line line 366-368; Needs rephrasing. Saying that "it was suggested" distances the discussion from the present results. The authors should discuss the results presented in the manuscript and therefore use present tense when referring to the results* **(Line 372)**

*Line 372-374; This needs to be rephrased. The statement implies that the authors were testing the hypothesis that smoking makes one younger. The foregoing discussion on aging does not appear to be linked to the finding on the age difference between the two groups. Although the difference was statistically significant, the difference does not appear to be clinically meaningful in the context of aging.* **(Line 378–381)**

*Line 380-381; "where" is not appropriately placed* **(Line 388)**
